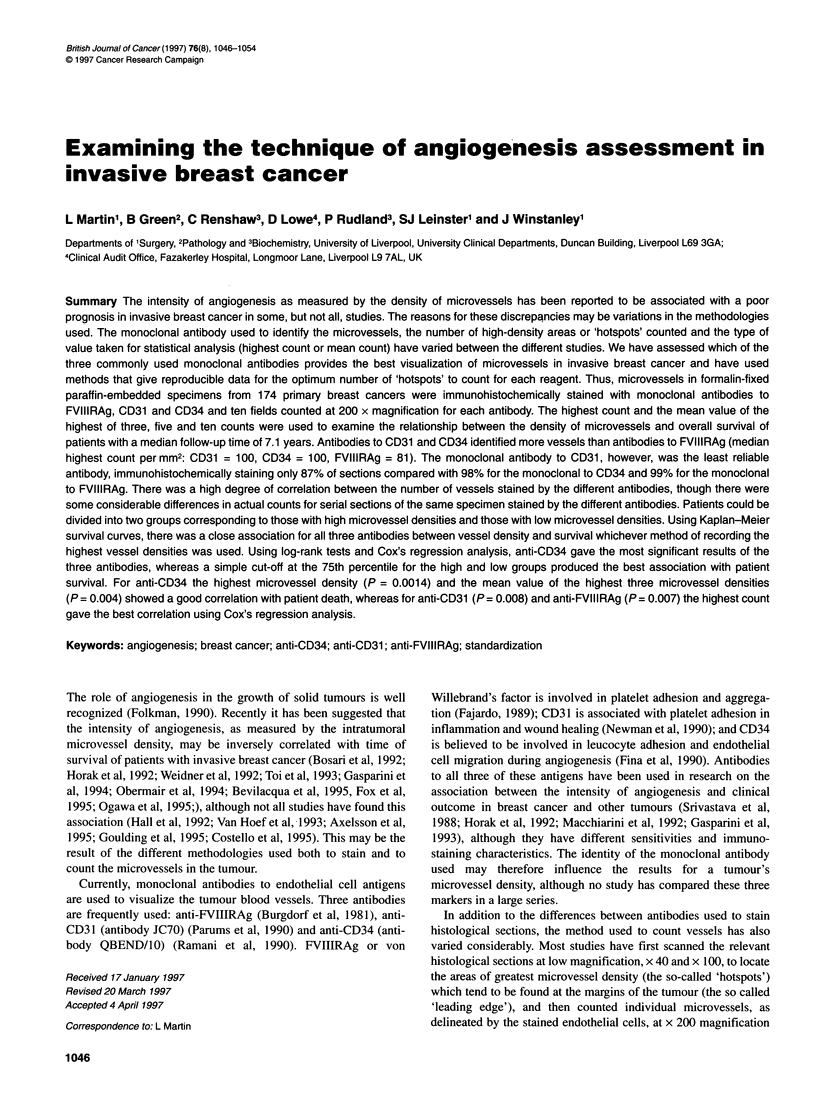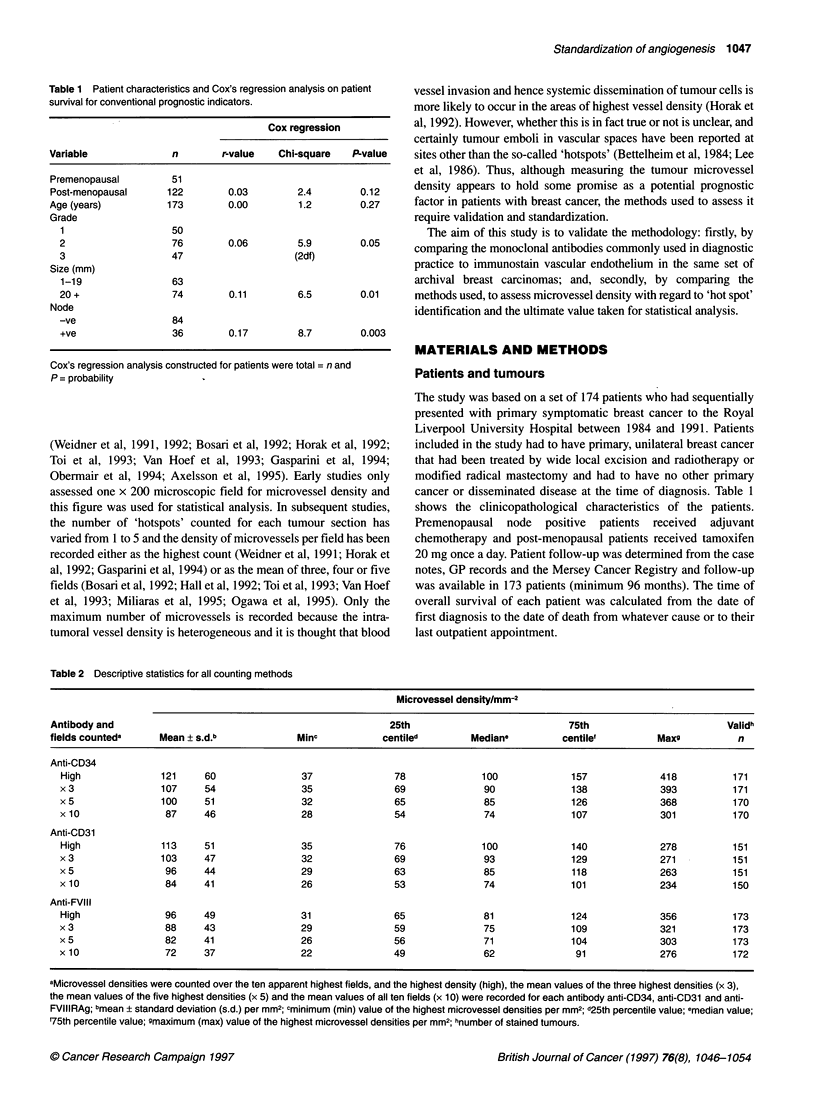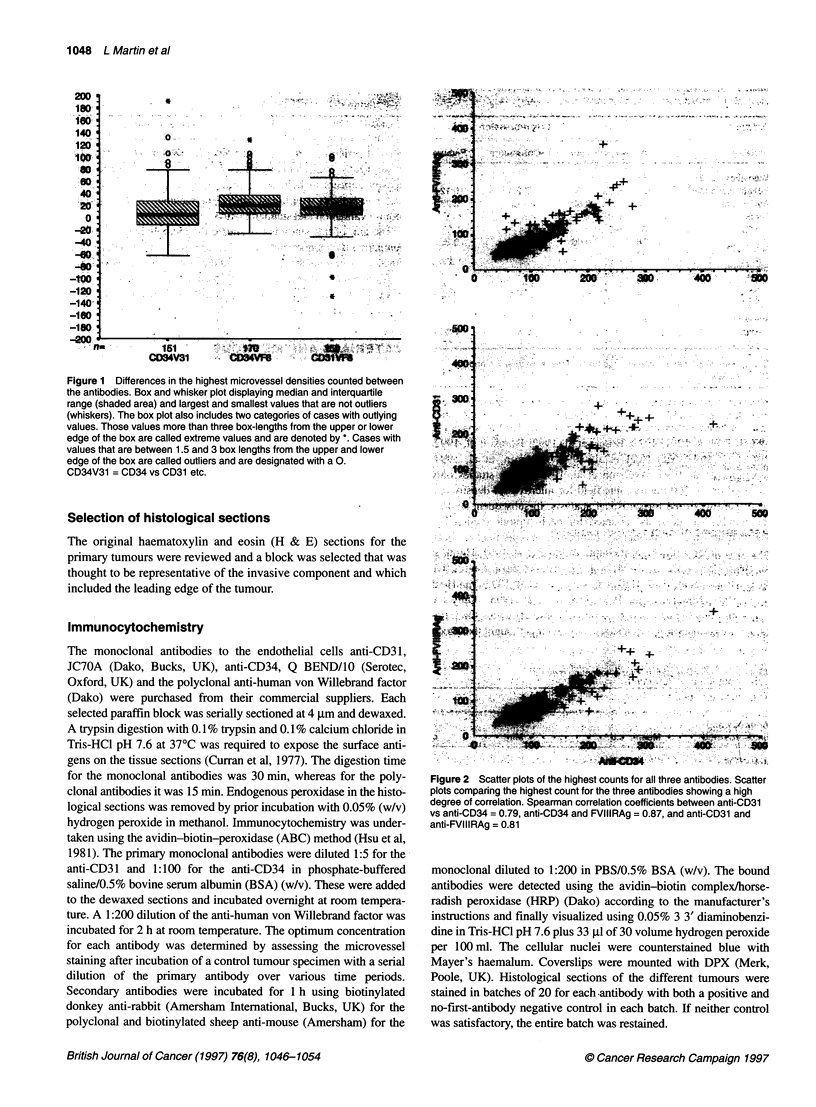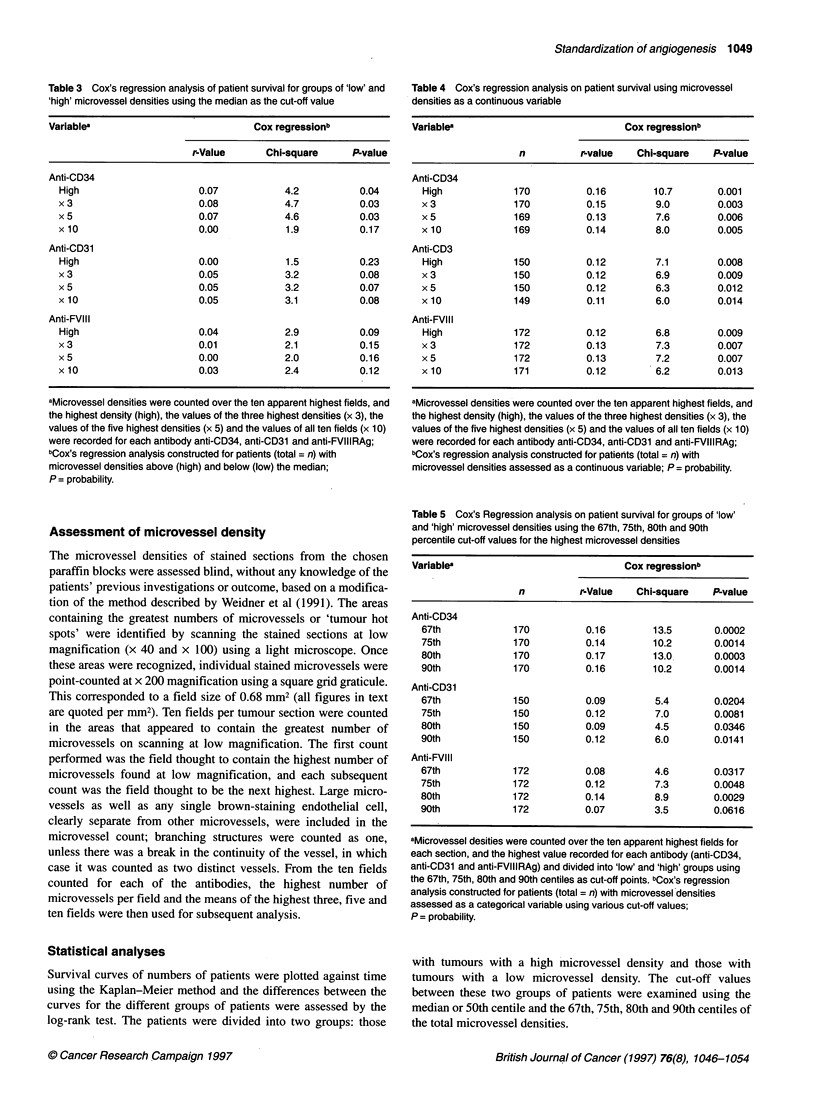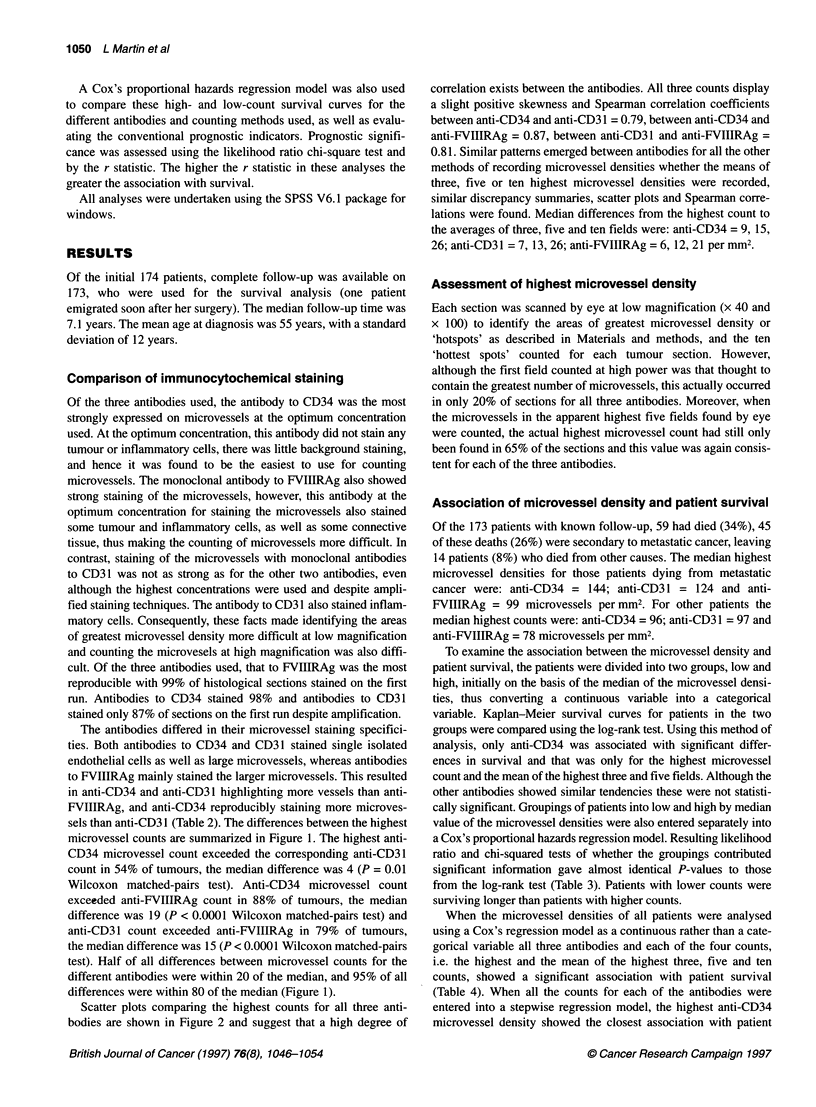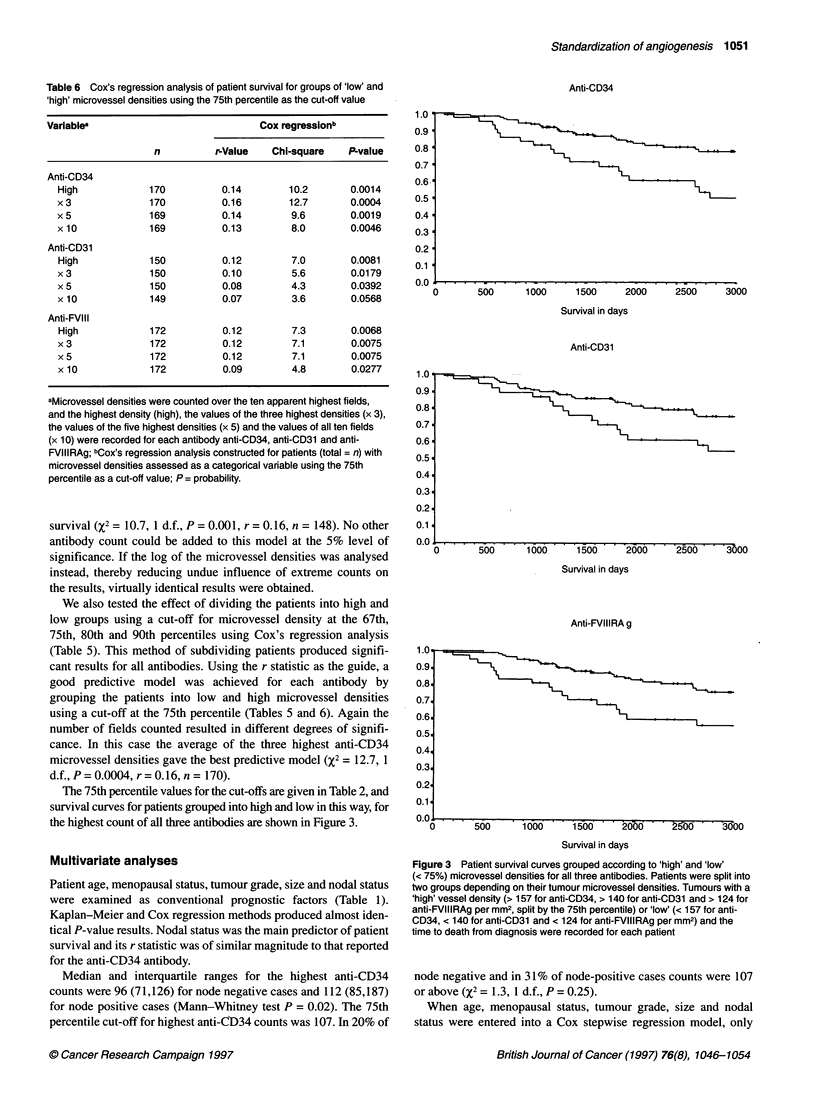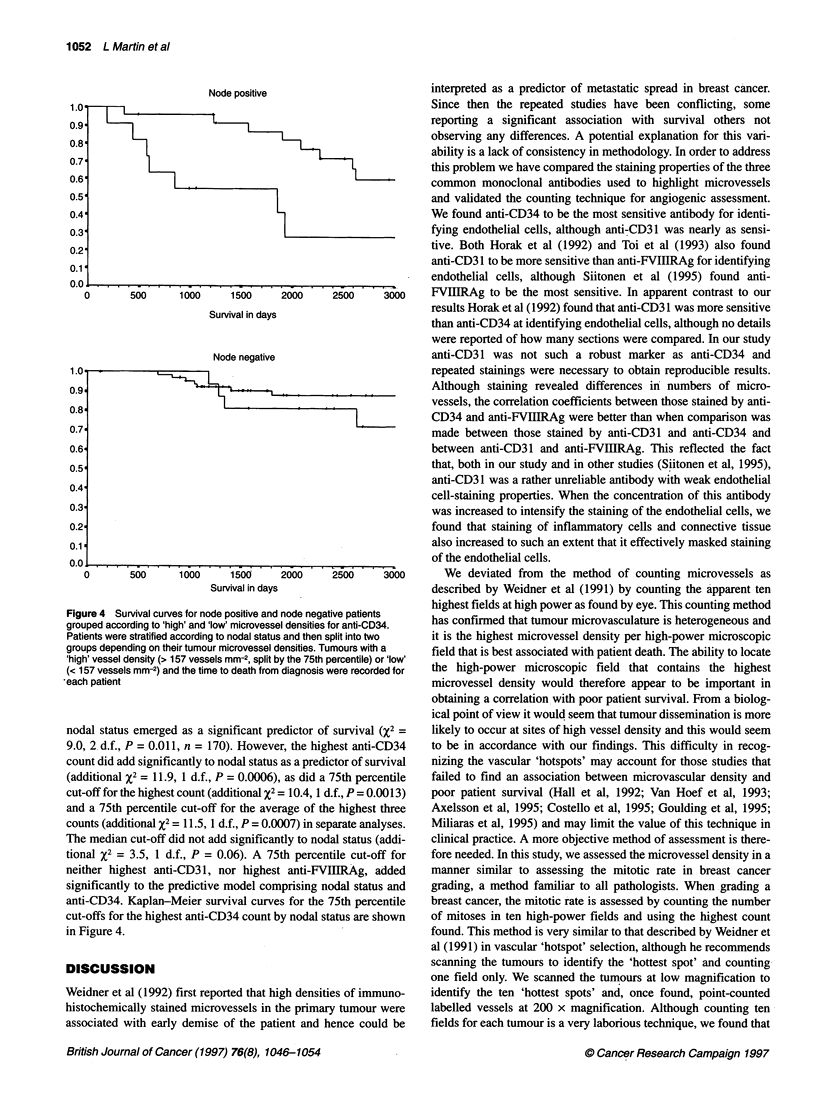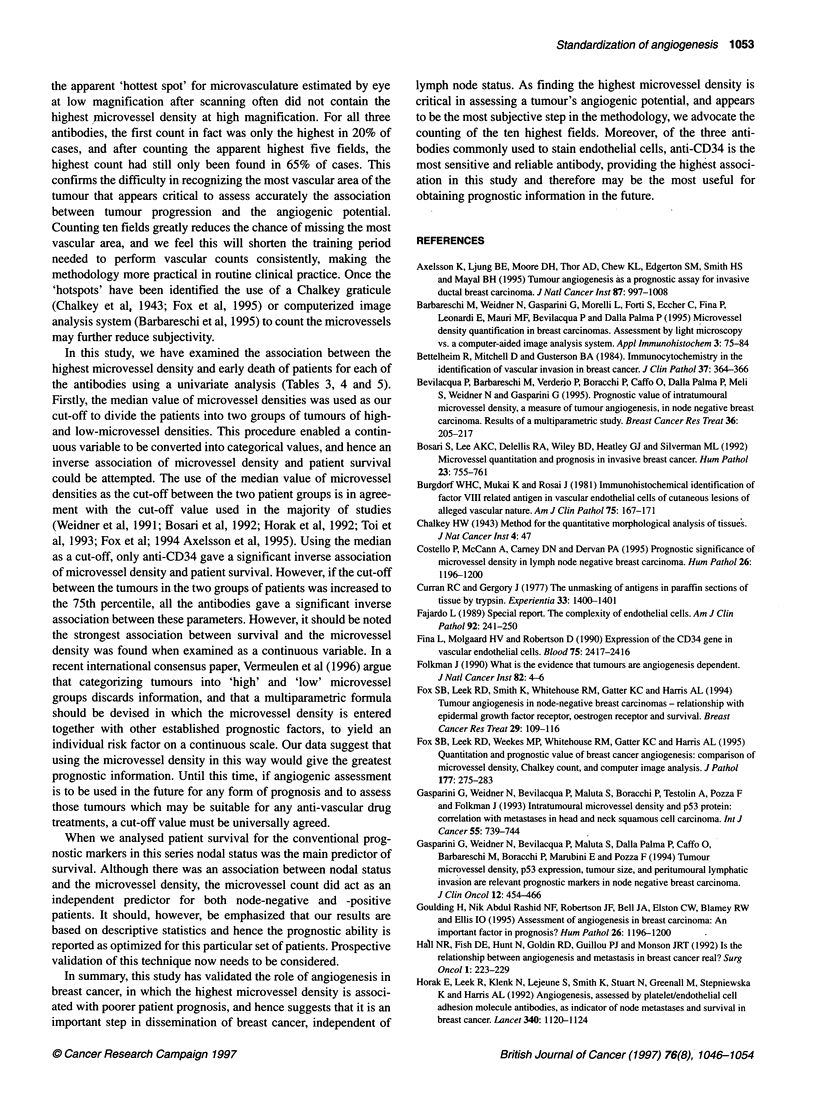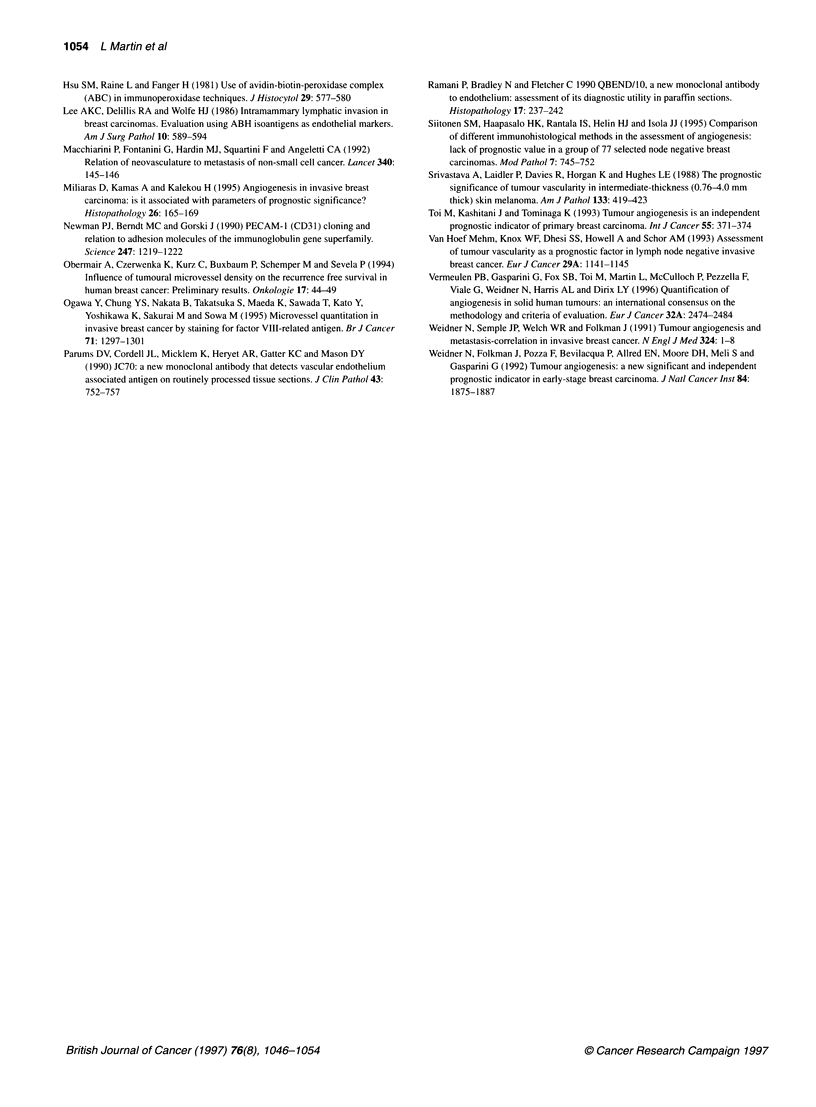# Examining the technique of angiogenesis assessment in invasive breast cancer.

**DOI:** 10.1038/bjc.1997.506

**Published:** 1997

**Authors:** L. Martin, B. Green, C. Renshaw, D. Lowe, P. Rudland, S. J. Leinster, J. Winstanley

**Affiliations:** Department of Surgery, University of Liverpool, UK.

## Abstract

The intensity of angiogenesis as measured by the density of microvessels has been reported to be associated with a poor prognosis in invasive breast cancer in some, but not all, studies. The reasons for these discrepancies may be variations in the methodologies used. The monoclonal antibody used to identify the microvessels, the number of high-density areas or 'hotspots' counted and the type of value taken for statistical analysis (highest count or mean count) have varied between the different studies. We have assessed which of the three commonly used monoclonal antibodies provides the best visualization of microvessels in invasive breast cancer and have used methods that give reproducible data for the optimum number of 'hotspots' to count for each reagent. Thus, microvessels in formalin-fixed paraffin-embedded specimens from 174 primary breast cancers were immunohistochemically stained with monoclonal antibodies to FVIIIRAg, CD31 and CD34 and ten fields counted at 200 x magnification for each antibody. The highest count and the mean value of the highest of three, five and ten counts were used to examine the relationship between the density of microvessels and overall survival of patients with a median follow-up time of 7.1 years. Antibodies to CD31 and CD34 identified more vessels than antibodies to FVIIIRAg (median highest count per mm2: CD31 = 100, CD34 = 100, FVIIIRAg = 81). The monoclonal antibody to CD31, however, was the least reliable antibody, immunohistochemically staining only 87% of sections compared with 98% for the monoclonal to CD34 and 99% for the monoclonal to FVIIIRAg. There was a high degree of correlation between the number of vessels stained by the different antibodies, though there were some considerable differences in actual counts for serial sections of the same specimen stained by the different antibodies. Patients could be divided into two groups corresponding to those with high microvessel densities and those with low microvessel densities. Using Kaplan-Meier survival curves, there was a close association for all three antibodies between vessel density and survival whichever method of recording the highest vessel densities was used. Using log-rank tests and Cox's regression analysis, anti-CD34 gave the most significant results of the three antibodies, whereas a simple cut-off at the 75th percentile for the high and low groups produced the best association with patient survival. For anti-CD34 the highest microvessel density (P = 0.0014) and the mean value of the highest three microvessel densities (P = 0.004) showed a good correlation with patient death, whereas for anti-CD31 (P = 0.008) and anti-FVIIIRAg (P = 0.007) the highest count gave the best correlation using Cox's regression analysis.


British Journal of Cancer (1997) **76**(8), 1046–1054
© 1997 Cancer Research Campaign

### Examining the technique of angiogenesis assessment in invasive breast cancer

**L Martin[1], B Green[2], C Renshaw[3], D Lowe[4], P Rudland[3], SJ Leinster[1] and J Winstanley[1]**

Departments of [1]Surgery, [2]Pathology and [3]Biochemistry, University of Liverpool, University Clinical Departments, Duncan Building, Liverpool L69 3GA; [4]Clinical Audit Office, Fazakerley Hospital, Longmoor Lane, Liverpool L9 7AL, UK

**Summary** The intensity of angiogenesis as measured by the density of microvessels has been reported to be associated with a poor prognosis in invasive breast cancer in some, but not all, studies. The reasons for these discrepancies may be variations in the methodologies used. The monoclonal antibody used to identify the microvessels, the number of high-density areas or 'hotspots' counted and the type of value taken for statistical analysis (highest count or mean count) have varied between the different studies. We have assessed which of the three commonly used monoclonal antibodies provides the best visualization of microvessels in invasive breast cancer and have used methods that give reproducible data for the optimum number of 'hotspots' to count for each reagent. Thus, microvessels in formalin-fixed paraffin-embedded specimens from 174 primary breast cancers were immunohistochemically stained with monoclonal antibodies to FVIIIRAg, CD31 and CD34 and ten fields counted at 200 × magnification for each antibody. The highest count and the mean value of the highest of three, five and ten counts were used to examine the relationship between the density of microvessels and overall survival of patients with a median follow-up time of 7.1 years. Antibodies to CD31 and CD34 identified more vessels than antibodies to FVIIIRAg (median highest count per mm$^2$: CD31 = 100, CD34 = 100, FVIIIRAg = 81). The monoclonal antibody to CD31, however, was the least reliable antibody, immunohistochemically staining only 87% of sections compared with 98% for the monoclonal to CD34 and 99% for the monoclonal to FVIIIRAg. There was a high degree of correlation between the number of vessels stained by the different antibodies, though there were some considerable differences in actual counts for serial sections of the same specimen stained by the different antibodies. Patients could be divided into two groups corresponding to those with high microvessel densities and those with low microvessel densities. Using Kaplan–Meier survival curves, there was a close association for all three antibodies between vessel density and survival whichever method of recording the highest vessel densities was used. Using log-rank tests and Cox's regression analysis, anti-CD34 gave the most significant results of the three antibodies, whereas a simple cut-off at the 75th percentile for the high and low groups produced the best association with patient survival. For anti-CD34 the highest microvessel density ($P$ = 0.0014) and the mean value of the highest three microvessel densities ($P$ = 0.004) showed a good correlation with patient death, whereas for anti-CD31 ($P$ = 0.008) and anti-FVIIIRAg ($P$ = 0.007) the highest count gave the best correlation using Cox's regression analysis.

**Keywords:** angiogenesis; breast cancer; anti-CD34; anti-CD31; anti-FVIIIRAg; standardization

The role of angiogenesis in the growth of solid tumours is well recognized (Folkman, 1990). Recently it has been suggested that the intensity of angiogenesis, as measured by the intratumoral microvessel density, may be inversely correlated with time of survival of patients with invasive breast cancer (Bosari et al, 1992; Horak et al, 1992; Weidner et al, 1992; Toi et al, 1993; Gasparini et al, 1994; Obermair et al, 1994; Bevilacqua et al, 1995, Fox et al, 1995; Ogawa et al, 1995;), although not all studies have found this association (Hall et al, 1992; Van Hoef et al, 1993; Axelsson et al, 1995; Goulding et al, 1995; Costello et al, 1995). This may be the result of the different methodologies used both to stain and to count the microvessels in the tumour.

Currently, monoclonal antibodies to endothelial cell antigens are used to visualize the tumour blood vessels. Three antibodies are frequently used: anti-FVIIIRAg (Burgdorf et al, 1981), anti-CD31 (antibody JC70) (Parums et al, 1990) and anti-CD34 (antibody QBEND/10) (Ramani et al, 1990). FVIIIRAg or von Willebrand's factor is involved in platelet adhesion and aggregation (Fajardo, 1989); CD31 is associated with platelet adhesion in inflammation and wound healing (Newman et al, 1990); and CD34 is believed to be involved in leucocyte adhesion and endothelial cell migration during angiogenesis (Fina et al, 1990). Antibodies to all three of these antigens have been used in research on the association between the intensity of angiogenesis and clinical outcome in breast cancer and other tumours (Srivastava et al, 1988; Horak et al, 1992; Macchiarini et al, 1992; Gasparini et al, 1993), although they have different sensitivities and immunostaining characteristics. The identity of the monoclonal antibody used may therefore influence the results for a tumour's microvessel density, although no study has compared these three markers in a large series.

In addition to the differences between antibodies used to stain histological sections, the method used to count vessels has also varied considerably. Most studies have first scanned the relevant histological sections at low magnification, × 40 and × 100, to locate the areas of greatest microvessel density (the so-called 'hotspots') which tend to be found at the margins of the tumour (the so called 'leading edge'), and then counted individual microvessels, as delineated by the stained endothelial cells, at × 200 magnification

*Received 17 January 1997*
*Revised 20 March 1997*
*Accepted 4 April 1997*

*Correspondence to:* L Martin

**Table 1** Patient characteristics and Cox's regression analysis on patient survival for conventional prognostic indicators.

| Variable | n | Cox regression *r*-value | Chi-square | *P*-value |
|---|---|---|---|---|
| Premenopausal | 51 | | | |
| Post-menopausal | 122 | 0.03 | 2.4 | 0.12 |
| Age (years) | 173 | 0.00 | 1.2 | 0.27 |
| Grade | | | | |
| 1 | 50 | | | |
| 2 | 76 | 0.06 | 5.9 | 0.05 |
| 3 | 47 | | (2df) | |
| Size (mm) | | | | |
| 1–19 | 63 | | | |
| 20 + | 74 | 0.11 | 6.5 | 0.01 |
| Node | | | | |
| –ve | 84 | | | |
| +ve | 36 | 0.17 | 8.7 | 0.003 |

Cox's regression analysis constructed for patients were total = *n* and *P* = probability

(Weidner et al, 1991, 1992; Bosari et al, 1992; Horak et al, 1992; Toi et al, 1993; Van Hoef et al, 1993; Gasparini et al, 1994; Obermair et al, 1994; Axelsson et al, 1995). Early studies only assessed one × 200 microscopic field for microvessel density and this figure was used for statistical analysis. In subsequent studies, the number of 'hotspots' counted for each tumour section has varied from 1 to 5 and the density of microvessels per field has been recorded either as the highest count (Weidner et al, 1991; Horak et al, 1992; Gasparini et al, 1994) or as the mean of three, four or five fields (Bosari et al, 1992; Hall et al, 1992; Toi et al, 1993; Van Hoef et al, 1993; Miliaras et al, 1995; Ogawa et al, 1995). Only the maximum number of microvessels is recorded because the intra-tumoral vessel density is heterogeneous and it is thought that blood vessel invasion and hence systemic dissemination of tumour cells is more likely to occur in the areas of highest vessel density (Horak et al, 1992). However, whether this is in fact true or not is unclear, and certainly tumour emboli in vascular spaces have been reported at sites other than the so-called 'hotspots' (Bettelheim et al, 1984; Lee et al, 1986). Thus, although measuring the tumour microvessel density appears to hold some promise as a potential prognostic factor in patients with breast cancer, the methods used to assess it require validation and standardization.

The aim of this study is to validate the methodology: firstly, by comparing the monoclonal antibodies commonly used in diagnostic practice to immunostain vascular endothelium in the same set of archival breast carcinomas; and, secondly, by comparing the methods used, to assess microvessel density with regard to 'hot spot' identification and the ultimate value taken for statistical analysis.

### MATERIALS AND METHODS

### Patients and tumours

The study was based on a set of 174 patients who had sequentially presented with primary symptomatic breast cancer to the Royal Liverpool University Hospital between 1984 and 1991. Patients included in the study had to have primary, unilateral breast cancer that had been treated by wide local excision and radiotherapy or modified radical mastectomy and had to have no other primary cancer or disseminated disease at the time of diagnosis. Table 1 shows the clinicopathological characteristics of the patients. Premenopausal node positive patients received adjuvant chemotherapy and post-menopausal patients received tamoxifen 20 mg once a day. Patient follow-up was determined from the case notes, GP records and the Mersey Cancer Registry and follow-up was available in 173 patients (minimum 96 months). The time of overall survival of each patient was calculated from the date of first diagnosis to the date of death from whatever cause or to their last outpatient appointment.

**Table 2** Descriptive statistics for all counting methods

| Antibody and fields counted[a] | Microvessel density/mm$^{-2}$ Mean ± s.d.[b] | | Min[c] | 25th centile[d] | Median[e] | 75th centile[f] | Max[g] | Valid[h] n |
|---|---|---|---|---|---|---|---|---|
| Anti-CD34 | | | | | | | | |
| High | 121 | 60 | 37 | 78 | 100 | 157 | 418 | 171 |
| × 3 | 107 | 54 | 35 | 69 | 90 | 138 | 393 | 171 |
| × 5 | 100 | 51 | 32 | 65 | 85 | 126 | 368 | 170 |
| × 10 | 87 | 46 | 28 | 54 | 74 | 107 | 301 | 170 |
| Anti-CD31 | | | | | | | | |
| High | 113 | 51 | 35 | 76 | 100 | 140 | 278 | 151 |
| × 3 | 103 | 47 | 32 | 69 | 93 | 129 | 271 | 151 |
| × 5 | 96 | 44 | 29 | 63 | 85 | 118 | 263 | 151 |
| × 10 | 84 | 41 | 26 | 53 | 74 | 101 | 234 | 150 |
| Anti-FVIII | | | | | | | | |
| High | 96 | 49 | 31 | 65 | 81 | 124 | 356 | 173 |
| × 3 | 88 | 43 | 29 | 59 | 75 | 109 | 321 | 173 |
| × 5 | 82 | 41 | 26 | 56 | 71 | 104 | 303 | 173 |
| × 10 | 72 | 37 | 22 | 49 | 62 | 91 | 276 | 172 |

[a]Microvessel densities were counted over the ten apparent highest fields, and the highest density (high), the mean values of the three highest densities (× 3), the mean values of the five highest densities (× 5) and the mean values of all ten fields (× 10) were recorded for each antibody anti-CD34, anti-CD31 and anti-FVIIIRAg; [b]mean ± standard deviation (s.d.) per mm$^2$; [c]minimum (min) value of the highest microvessel densities per mm$^2$; [d]25th percentile value; [e]median value; [f]75th percentile value; [g]maximum (max) value of the highest microvessel densities per mm$^2$; [h]number of stained tumours.

© Cancer Research Campaign 1997

**Figure 1** Differences in the highest microvessel densities counted between the antibodies. Box and whisker plot displaying median and interquartile range (shaded area) and largest and smallest values that are not outliers (whiskers). The box plot also includes two categories of cases with outlying values. Those values more than three box-lengths from the upper or lower edge of the box are called extreme values and are denoted by *. Cases with values that are between 1.5 and 3 box lengths from the upper and lower edge of the box are called outliers and are designated with a O. CD34V31 = CD34 vs CD31 etc.

### Selection of histological sections

The original haematoxylin and eosin (H & E) sections for the primary tumours were reviewed and a block was selected that was thought to be representative of the invasive component and which included the leading edge of the tumour.

### Immunocytochemistry

The monoclonal antibodies to the endothelial cells anti-CD31, JC70A (Dako, Bucks, UK), anti-CD34, Q BEND/10 (Serotec, Oxford, UK) and the polyclonal anti-human von Willebrand factor (Dako) were purchased from their commercial suppliers. Each selected paraffin block was serially sectioned at 4 μm and dewaxed. A trypsin digestion with 0.1% trypsin and 0.1% calcium chloride in Tris-HCl pH 7.6 at 37°C was required to expose the surface antigens on the tissue sections (Curran et al, 1977). The digestion time for the monoclonal antibodies was 30 min, whereas for the polyclonal antibodies it was 15 min. Endogenous peroxidase in the histological sections was removed by prior incubation with 0.05% (w/v) hydrogen peroxide in methanol. Immunocytochemistry was undertaken using the avidin–biotin–peroxidase (ABC) method (Hsu et al, 1981). The primary monoclonal antibodies were diluted 1:5 for the anti-CD31 and 1:100 for the anti-CD34 in phosphate-buffered saline/0.5% bovine serum albumin (BSA) (w/v). These were added to the dewaxed sections and incubated overnight at room temperature. A 1:200 dilution of the anti-human von Willebrand factor was incubated for 2 h at room temperature. The optimum concentration for each antibody was determined by assessing the microvessel staining after incubation of a control tumour specimen with a serial dilution of the primary antibody over various time periods. Secondary antibodies were incubated for 1 h using biotinylated donkey anti-rabbit (Amersham International, Bucks, UK) for the polyclonal and biotinylated sheep anti-mouse (Amersham) for the

**Figure 2** Scatter plots of the highest counts for all three antibodies. Scatter plots comparing the highest count for the three antibodies showing a high degree of correlation. Spearman correlation coefficients between anti-CD31 vs anti-CD34 = 0.79, anti-CD34 and FVIIIRAg = 0.87, and anti-CD31 and anti-FVIIIRAg = 0.81

monoclonal diluted to 1:200 in PBS/0.5% BSA (w/v). The bound antibodies were detected using the avidin–biotin complex/horseradish peroxidase (HRP) (Dako) according to the manufacturer's instructions and finally visualized using 0.05% 3 3′ diaminobenzidine in Tris-HCl pH 7.6 plus 33 μl of 30 volume hydrogen peroxide per 100 ml. The cellular nuclei were counterstained blue with Mayer's haemalum. Coverslips were mounted with DPX (Merk, Poole, UK). Histological sections of the different tumours were stained in batches of 20 for each antibody with both a positive and no-first-antibody negative control in each batch. If neither control was satisfactory, the entire batch was restained.

*© Cancer Research Campaign 1997*

**Table 3** Cox's regression analysis of patient survival for groups of 'low' and 'high' microvessel densities using the median as the cut-off value

| Variable[a] | Cox regression[b] | | |
|---|---|---|---|
| | *r*-Value | Chi-square | *P*-value |
| Anti-CD34 | | | |
| High | 0.07 | 4.2 | 0.04 |
| × 3 | 0.08 | 4.7 | 0.03 |
| × 5 | 0.07 | 4.6 | 0.03 |
| × 10 | 0.00 | 1.9 | 0.17 |
| Anti-CD31 | | | |
| High | 0.00 | 1.5 | 0.23 |
| × 3 | 0.05 | 3.2 | 0.08 |
| × 5 | 0.05 | 3.2 | 0.07 |
| × 10 | 0.05 | 3.1 | 0.08 |
| Anti-FVIII | | | |
| High | 0.04 | 2.9 | 0.09 |
| × 3 | 0.01 | 2.1 | 0.15 |
| × 5 | 0.00 | 2.0 | 0.16 |
| × 10 | 0.03 | 2.4 | 0.12 |

[a]Microvessel densities were counted over the ten apparent highest fields, and the highest density (high), the values of the three highest densities (× 3), the values of the five highest densities (× 5) and the values of all ten fields (× 10) were recorded for each antibody anti-CD34, anti-CD31 and anti-FVIIIRAg; [b]Cox's regression analysis constructed for patients (total = *n*) with microvessel densities above (high) and below (low) the median; *P* = probability.

**Table 4** Cox's regression analysis on patient survival using microvessel densities as a continuous variable

| Variable[a] | | Cox regression[b] | | |
|---|---|---|---|---|
| | *n* | *r*-value | Chi-square | *P*-value |
| Anti-CD34 | | | | |
| High | 170 | 0.16 | 10.7 | 0.001 |
| × 3 | 170 | 0.15 | 9.0 | 0.003 |
| × 5 | 169 | 0.13 | 7.6 | 0.006 |
| × 10 | 169 | 0.14 | 8.0 | 0.005 |
| Anti-CD3 | | | | |
| High | 150 | 0.12 | 7.1 | 0.008 |
| × 3 | 150 | 0.12 | 6.9 | 0.009 |
| × 5 | 150 | 0.12 | 6.3 | 0.012 |
| × 10 | 149 | 0.11 | 6.0 | 0.014 |
| Anti-FVIII | | | | |
| High | 172 | 0.12 | 6.8 | 0.009 |
| × 3 | 172 | 0.13 | 7.3 | 0.007 |
| × 5 | 172 | 0.13 | 7.2 | 0.007 |
| × 10 | 171 | 0.12 | 6.2 | 0.013 |

[a]Microvessel densities were counted over the ten apparent highest fields, and the highest density (high), the values of the three highest densities (× 3), the values of the five highest densities (× 5) and the values of all ten fields (× 10) were recorded for each antibody anti-CD34, anti-CD31 and anti-FVIIIRAg; [b]Cox's regression analysis constructed for patients (total = *n*) with microvessel densities assessed as a continuous variable; *P* = probability.

### Assessment of microvessel density

The microvessel densities of stained sections from the chosen paraffin blocks were assessed blind, without any knowledge of the patients' previous investigations or outcome, based on a modification of the method described by Weidner et al (1991). The areas containing the greatest numbers of microvessels or 'tumour hot spots' were identified by scanning the stained sections at low magnification (× 40 and × 100) using a light microscope. Once these areas were recognized, individual stained microvessels were point-counted at × 200 magnification using a square grid graticule. This corresponded to a field size of 0.68 $mm^2$ (all figures in text are quoted per $mm^2$). Ten fields per tumour section were counted in the areas that appeared to contain the greatest number of microvessels on scanning at low magnification. The first count performed was the field thought to contain the highest number of microvessels found at low magnification, and each subsequent count was the field thought to be the next highest. Large microvessels as well as any single brown-staining endothelial cell, clearly separate from other microvessels, were included in the microvessel count; branching structures were counted as one, unless there was a break in the continuity of the vessel, in which case it was counted as two distinct vessels. From the ten fields counted for each of the antibodies, the highest number of microvessels per field and the means of the highest three, five and ten fields were then used for subsequent analysis.

### Statistical analyses

Survival curves of numbers of patients were plotted against time using the Kaplan–Meier method and the differences between the curves for the different groups of patients were assessed by the log-rank test. The patients were divided into two groups: those with tumours with a high microvessel density and those with tumours with a low microvessel density. The cut-off values between these two groups of patients were examined using the median or 50th centile and the 67th, 75th, 80th and 90th centiles of the total microvessel densities.

**Table 5** Cox's Regression analysis on patient survival for groups of 'low' and 'high' microvessel densities using the 67th, 75th, 80th and 90th percentile cut-off values for the highest microvessel densities

| Variable[a] | | Cox regression[b] | | |
|---|---|---|---|---|
| | *n* | *r*-Value | Chi-square | *P*-value |
| Anti-CD34 | | | | |
| 67th | 170 | 0.16 | 13.5 | 0.0002 |
| 75th | 170 | 0.14 | 10.2 | 0.0014 |
| 80th | 170 | 0.17 | 13.0 | 0.0003 |
| 90th | 170 | 0.16 | 10.2 | 0.0014 |
| Anti-CD31 | | | | |
| 67th | 150 | 0.09 | 5.4 | 0.0204 |
| 75th | 150 | 0.12 | 7.0 | 0.0081 |
| 80th | 150 | 0.09 | 4.5 | 0.0346 |
| 90th | 150 | 0.12 | 6.0 | 0.0141 |
| Anti-FVIII | | | | |
| 67th | 172 | 0.08 | 4.6 | 0.0317 |
| 75th | 172 | 0.12 | 7.3 | 0.0048 |
| 80th | 172 | 0.14 | 8.9 | 0.0029 |
| 90th | 172 | 0.07 | 3.5 | 0.0616 |

[a]Microvessel desities were counted over the ten apparent highest fields for each section, and the highest value recorded for each antibody (anti-CD34, anti-CD31 and anti-FVIIIRAg) and divided into 'low' and 'high' groups using the 67th, 75th, 80th and 90th centiles as cut-off points. [b]Cox's regression analysis constructed for patients (total = *n*) with microvessel densities assessed as a categorical variable using various cut-off values; *P* = probability.

© Cancer Research Campaign 1997

A Cox's proportional hazards regression model was also used to compare these high- and low-count survival curves for the different antibodies and counting methods used, as well as evaluating the conventional prognostic indicators. Prognostic significance was assessed using the likelihood ratio chi-square test and by the *r* statistic. The higher the *r* statistic in these analyses the greater the association with survival.

All analyses were undertaken using the SPSS V6.1 package for windows.

### RESULTS

Of the initial 174 patients, complete follow-up was available on 173, who were used for the survival analysis (one patient emigrated soon after her surgery). The median follow-up time was 7.1 years. The mean age at diagnosis was 55 years, with a standard deviation of 12 years.

### Comparison of immunocytochemical staining

Of the three antibodies used, the antibody to CD34 was the most strongly expressed on microvessels at the optimum concentration used. At the optimum concentration, this antibody did not stain any tumour or inflammatory cells, there was little background staining, and hence it was found to be the easiest to use for counting microvessels. The monoclonal antibody to FVIIIRAg also showed strong staining of the microvessels, however, this antibody at the optimum concentration for staining the microvessels also stained some tumour and inflammatory cells, as well as some connective tissue, thus making the counting of microvessels more difficult. In contrast, staining of the microvessels with monoclonal antibodies to CD31 was not as strong as for the other two antibodies, even although the highest concentrations were used and despite amplified staining techniques. The antibody to CD31 also stained inflammatory cells. Consequently, these facts made identifying the areas of greatest microvessel density more difficult at low magnification and counting the microvesels at high magnification was also difficult. Of the three antibodies used, that to FVIIIRAg was the most reproducible with 99% of histological sections stained on the first run. Antibodies to CD34 stained 98% and antibodies to CD31 stained only 87% of sections on the first run despite amplification.

The antibodies differed in their microvessel staining specificities. Both antibodies to CD34 and CD31 stained single isolated endothelial cells as well as large microvessels, whereas antibodies to FVIIIRAg mainly stained the larger microvessels. This resulted in anti-CD34 and anti-CD31 highlighting more vessels than anti-FVIIIRAg, and anti-CD34 reproducibly staining more microvessels than anti-CD31 (Table 2). The differences between the highest microvessel counts are summarized in Figure 1. The highest anti-CD34 microvessel count exceeded the corresponding anti-CD31 count in 54% of tumours, the median difference was 4 ($P = 0.01$ Wilcoxon matched-pairs test). Anti-CD34 microvessel count exceeded anti-FVIIIRAg count in 88% of tumours, the median difference was 19 ($P < 0.0001$ Wilcoxon matched-pairs test) and anti-CD31 count exceeded anti-FVIIIRAg in 79% of tumours, the median difference was 15 ($P < 0.0001$ Wilcoxon matched-pairs test). Half of all differences between microvessel counts for the different antibodies were within 20 of the median, and 95% of all differences were within 80 of the median (Figure 1).

Scatter plots comparing the highest counts for all three antibodies are shown in Figure 2 and suggest that a high degree of correlation exists between the antibodies. All three counts display a slight positive skewness and Spearman correlation coefficients between anti-CD34 and anti-CD31 = 0.79, between anti-CD34 and anti-FVIIIRAg = 0.87, between anti-CD31 and anti-FVIIIRAg = 0.81. Similar patterns emerged between antibodies for all the other methods of recording microvessel densities whether the means of three, five or ten highest microvessel densities were recorded, similar discrepancy summaries, scatter plots and Spearman correlations were found. Median differences from the highest count to the averages of three, five and ten fields were: anti-CD34 = 9, 15, 26; anti-CD31 = 7, 13, 26; anti-FVIIIRAg = 6, 12, 21 per mm$^2$.

### Assessment of highest microvessel density

Each section was scanned by eye at low magnification (× 40 and × 100) to identify the areas of greatest microvessel density or 'hotspots' as described in Materials and methods, and the ten 'hottest spots' counted for each tumour section. However, although the first field counted at high power was that thought to contain the greatest number of microvessels, this actually occurred in only 20% of sections for all three antibodies. Moreover, when the microvessels in the apparent highest five fields found by eye were counted, the actual highest microvessel count had still only been found in 65% of the sections and this value was again consistent for each of the three antibodies.

### Association of microvessel density and patient survival

Of the 173 patients with known follow-up, 59 had died (34%), 45 of these deaths (26%) were secondary to metastatic cancer, leaving 14 patients (8%) who died from other causes. The median highest microvessel densities for those patients dying from metastatic cancer were: anti-CD34 = 144; anti-CD31 = 124 and anti-FVIIIRAg = 99 microvessels per mm$^2$. For other patients the median highest counts were: anti-CD34 = 96; anti-CD31 = 97 and anti-FVIIIRAg = 78 microvessels per mm$^2$.

To examine the association between the microvessel density and patient survival, the patients were divided into two groups, low and high, initially on the basis of the median of the microvessel densities, thus converting a continuous variable into a categorical variable. Kaplan–Meier survival curves for patients in the two groups were compared using the log-rank test. Using this method of analysis, only anti-CD34 was associated with significant differences in survival and that was only for the highest microvessel count and the mean of the highest three and five fields. Although the other antibodies showed similar tendencies these were not statistically significant. Groupings of patients into low and high by median value of the microvessel densities were also entered separately into a Cox's proportional hazards regression model. Resulting likelihood ratio and chi-squared tests of whether the groupings contributed significant information gave almost identical *P*-values to those from the log-rank test (Table 3). Patients with lower counts were surviving longer than patients with higher counts.

When the microvessel densities of all patients were analysed using a Cox's regression model as a continuous rather than a categorical variable all three antibodies and each of the four counts, i.e. the highest and the mean of the highest three, five and ten counts, showed a significant association with patient survival (Table 4). When all the counts for each of the antibodies were entered into a stepwise regression model, the highest anti-CD34 microvessel density showed the closest association with patient

© Cancer Research Campaign 1997

**Table 6** Cox's regression analysis of patient survival for groups of 'low' and 'high' microvessel densities using the 75th percentile as the cut-off value

| Variable[a] | | Cox regression[b] | | |
|---|---|---|---|---|
| | *n* | *r*-Value | Chi-square | *P*-value |
| Anti-CD34 | | | | |
| High | 170 | 0.14 | 10.2 | 0.0014 |
| × 3 | 170 | 0.16 | 12.7 | 0.0004 |
| × 5 | 169 | 0.14 | 9.6 | 0.0019 |
| × 10 | 169 | 0.13 | 8.0 | 0.0046 |
| Anti-CD31 | | | | |
| High | 150 | 0.12 | 7.0 | 0.0081 |
| × 3 | 150 | 0.10 | 5.6 | 0.0179 |
| × 5 | 150 | 0.08 | 4.3 | 0.0392 |
| × 10 | 149 | 0.07 | 3.6 | 0.0568 |
| Anti-FVIII | | | | |
| High | 172 | 0.12 | 7.3 | 0.0068 |
| × 3 | 172 | 0.12 | 7.1 | 0.0075 |
| × 5 | 172 | 0.12 | 7.1 | 0.0075 |
| × 10 | 172 | 0.09 | 4.8 | 0.0277 |

[a]Microvessel densities were counted over the ten apparent highest fields, and the highest density (high), the values of the three highest densities (× 3), the values of the five highest densities (× 5) and the values of all ten fields (× 10) were recorded for each antibody anti-CD34, anti-CD31 and anti-FVIIIRAg; [b]Cox's regression analysis constructed for patients (total = *n*) with microvessel densities assessed as a categorical variable using the 75th percentile as a cut-off value; *P* = probability.

survival ($\chi^2$ = 10.7, 1 d.f., $P$ = 0.001, $r$ = 0.16, $n$ = 148). No other antibody count could be added to this model at the 5% level of significance. If the log of the microvessel densities was analysed instead, thereby reducing undue influence of extreme counts on the results, virtually identical results were obtained.

We also tested the effect of dividing the patients into high and low groups using a cut-off for microvessel density at the 67th, 75th, 80th and 90th percentiles using Cox's regression analysis (Table 5). This method of subdividing patients produced significant results for all antibodies. Using the $r$ statistic as the guide, a good predictive model was achieved for each antibody by grouping the patients into low and high microvessel densities using a cut-off at the 75th percentile (Tables 5 and 6). Again the number of fields counted resulted in different degrees of significance. In this case the average of the three highest anti-CD34 microvessel densities gave the best predictive model ($\chi^2$ = 12.7, 1 d.f., $P$ = 0.0004, $r$ = 0.16, $n$ = 170).

The 75th percentile values for the cut-offs are given in Table 2, and survival curves for patients grouped into high and low in this way, for the highest count of all three antibodies are shown in Figure 3.

### Multivariate analyses

Patient age, menopausal status, tumour grade, size and nodal status were examined as conventional prognostic factors (Table 1). Kaplan–Meier and Cox regression methods produced almost identical $P$-value results. Nodal status was the main predictor of patient survival and its $r$ statistic was of similar magnitude to that reported for the anti-CD34 antibody.

Median and interquartile ranges for the highest anti-CD34 counts were 96 (71,126) for node negative cases and 112 (85,187) for node positive cases (Mann–Whitney test $P$ = 0.02). The 75th percentile cut-off for highest anti-CD34 counts was 107. In 20% of node negative and in 31% of node-positive cases counts were 107 or above ($\chi^2$ = 1.3, 1 d.f., $P$ = 0.25).

**Figure 3** Patient survival curves grouped according to 'high' and 'low' (< 75%) microvessel densities for all three antibodies. Patients were split into two groups depending on their tumour microvessel densities. Tumours with a 'high' vessel density (> 157 for anti-CD34, > 140 for anti-CD31 and > 124 for anti-FVIIIRAg per mm², split by the 75th percentile) or 'low' (< 157 for anti-CD34, < 140 for anti-CD31 and < 124 for anti-FVIIIRAg per mm²) and the time to death from diagnosis were recorded for each patient

When age, menopausal status, tumour grade, size and nodal status were entered into a Cox stepwise regression model, only

**Figure 4** Survival curves for node positive and node negative patients grouped according to 'high' and 'low' microvessel densities for anti-CD34. Patients were stratified according to nodal status and then split into two groups depending on their tumour microvessel densities. Tumours with a 'high' vessel density (> 157 vessels $mm^{-2}$, split by the 75th percentile) or 'low' (< 157 vessels $mm^{-2}$) and the time to death from diagnosis were recorded for each patient nodal status emerged as a significant predictor of survival ($\chi^2$ = 9.0, 2 d.f., $P$ = 0.011, $n$ = 170). However, the highest anti-CD34 count did add significantly to nodal status as a predictor of survival (additional $\chi^2$ = 11.9, 1 d.f., $P$ = 0.0006), as did a 75th percentile cut-off for the highest count (additional $\chi^2$ = 10.4, 1 d.f., $P$ = 0.0013) and a 75th percentile cut-off for the average of the highest three counts (additional $\chi^2$ = 11.5, 1 d.f., $P$ = 0.0007) in separate analyses. The median cut-off did not add significantly to nodal status (additional $\chi^2$ = 3.5, 1 d.f., $P$ = 0.06). A 75th percentile cut-off for neither highest anti-CD31, nor highest anti-FVIIIRAg, added significantly to the predictive model comprising nodal status and anti-CD34. Kaplan–Meier survival curves for the 75th percentile cut-offs for the highest anti-CD34 count by nodal status are shown in Figure 4.

### DISCUSSION

Weidner et al (1992) first reported that high densities of immunohistochemically stained microvessels in the primary tumour were associated with early demise of the patient and hence could be interpreted as a predictor of metastatic spread in breast cancer. Since then the repeated studies have been conflicting, some reporting a significant association with survival others not observing any differences. A potential explanation for this variability is a lack of consistency in methodology. In order to address this problem we have compared the staining properties of the three common monoclonal antibodies used to highlight microvessels and validated the counting technique for angiogenic assessment. We found anti-CD34 to be the most sensitive antibody for identifying endothelial cells, although anti-CD31 was nearly as sensitive. Both Horak et al (1992) and Toi et al (1993) also found anti-CD31 to be more sensitive than anti-FVIIIRAg for identifying endothelial cells, although Siitonen et al (1995) found anti-FVIIIRAg to be the most sensitive. In apparent contrast to our results Horak et al (1992) found that anti-CD31 was more sensitive than anti-CD34 at identifying endothelial cells, although no details were reported of how many sections were compared. In our study anti-CD31 was not such a robust marker as anti-CD34 and repeated stainings were necessary to obtain reproducible results. Although staining revealed differences in numbers of microvessels, the correlation coefficients between those stained by anti-CD34 and anti-FVIIIRAg were better than when comparison was made between those stained by anti-CD31 and anti-CD34 and between anti-CD31 and anti-FVIIIRAg. This reflected the fact that, both in our study and in other studies (Siitonen et al, 1995), anti-CD31 was a rather unreliable antibody with weak endothelial cell-staining properties. When the concentration of this antibody was increased to intensify the staining of the endothelial cells, we found that staining of inflammatory cells and connective tissue also increased to such an extent that it effectively masked staining of the endothelial cells.

We deviated from the method of counting microvessels as described by Weidner et al (1991) by counting the apparent ten highest fields at high power as found by eye. This counting method has confirmed that tumour microvasculature is heterogeneous and it is the highest microvessel density per high-power microscopic field that is best associated with patient death. The ability to locate the high-power microscopic field that contains the highest microvessel density would therefore appear to be important in obtaining a correlation with poor patient survival. From a biological point of view it would seem that tumour dissemination is more likely to occur at sites of high vessel density and this would seem to be in accordance with our findings. This difficulty in recognizing the vascular 'hotspots' may account for those studies that failed to find an association between microvascular density and poor patient survival (Hall et al, 1992; Van Hoef et al, 1993; Axelsson et al, 1995; Costello et al, 1995; Goulding et al, 1995; Miliaras et al, 1995) and may limit the value of this technique in clinical practice. A more objective method of assessment is therefore needed. In this study, we assessed the microvessel density in a manner similar to assessing the mitotic rate in breast cancer grading, a method familiar to all pathologists. When grading a breast cancer, the mitotic rate is assessed by counting the number of mitoses in ten high-power fields and using the highest count found. This method is very similar to that described by Weidner et al (1991) in vascular 'hotspot' selection, although he recommends scanning the tumours to identify the 'hottest spot' and counting one field only. We scanned the tumours at low magnification to identify the ten 'hottest spots' and, once found, point-counted labelled vessels at 200 × magnification. Although counting ten fields for each tumour is a very laborious technique, we found that

© Cancer Research Campaign 1997

the apparent 'hottest spot' for microvasculature estimated by eye at low magnification after scanning often did not contain the highest microvessel density at high magnification. For all three antibodies, the first count in fact was only the highest in 20% of cases, and after counting the apparent highest five fields, the highest count had still only been found in 65% of cases. This confirms the difficulty in recognizing the most vascular area of the tumour that appears critical to assess accurately the association between tumour progression and the angiogenic potential. Counting ten fields greatly reduces the chance of missing the most vascular area, and we feel this will shorten the training period needed to perform vascular counts consistently, making the methodology more practical in routine clinical practice. Once the 'hotspots' have been identified the use of a Chalkey graticule (Chalkey et al, 1943; Fox et al, 1995) or computerized image analysis system (Barbareschi et al, 1995) to count the microvessels may further reduce subjectivity.

In this study, we have examined the association between the highest microvessel density and early death of patients for each of the antibodies using a univariate analysis (Tables 3, 4 and 5). Firstly, the median value of microvessel densities was used as our cut-off to divide the patients into two groups of tumours of high- and low-microvessel densities. This procedure enabled a continuous variable to be converted into categorical values, and hence an inverse association of microvessel density and patient survival could be attempted. The use of the median value of microvessel densities as the cut-off between the two patient groups is in agreement with the cut-off value used in the majority of studies (Weidner et al, 1991; Bosari et al, 1992; Horak et al, 1992; Toi et al, 1993; Fox et al; 1994 Axelsson et al, 1995). Using the median as a cut-off, only anti-CD34 gave a significant inverse association of microvessel density and patient survival. However, if the cut-off between the tumours in the two groups of patients was increased to the 75th percentile, all the antibodies gave a significant inverse association between these parameters. However, it should be noted the strongest association between survival and the microvessel density was found when examined as a continuous variable. In a recent international consensus paper, Vermeulen et al (1996) argue that categorizing tumours into 'high' and 'low' microvessel groups discards information, and that a multiparametric formula should be devised in which the microvessel density is entered together with other established prognostic factors, to yield an individual risk factor on a continuous scale. Our data suggest that using the microvessel density in this way would give the greatest prognostic information. Until this time, if angiogenic assessment is to be used in the future for any form of prognosis and to assess those tumours which may be suitable for any anti-vascular drug treatments, a cut-off value must be universally agreed.

When we analysed patient survival for the conventional prognostic markers in this series nodal status was the main predictor of survival. Although there was an association between nodal status and the microvessel density, the microvessel count did act as an independent predictor for both node-negative and -positive patients. It should, however, be emphasized that our results are based on descriptive statistics and hence the prognostic ability is reported as optimized for this particular set of patients. Prospective validation of this technique now needs to be considered.

In summary, this study has validated the role of angiogenesis in breast cancer, in which the highest microvessel density is associated with poorer patient prognosis, and hence suggests that it is an important step in dissemination of breast cancer, independent of lymph node status. As finding the highest microvessel density is critical in assessing a tumour's angiogenic potential, and appears to be the most subjective step in the methodology, we advocate the counting of the ten highest fields. Moreover, of the three antibodies commonly used to stain endothelial cells, anti-CD34 is the most sensitive and reliable antibody, providing the highest association in this study and therefore may be the most useful for obtaining prognostic information in the future.

### REFERENCES

Axelsson K, Ljung BE, Moore DH, Thor AD, Chew KL, Edgerton SM, Smith HS and Mayal BH (1995) Tumour angiogenesis as a prognostic assay for invasive ductal breast carcinoma. *J Natl Cancer Inst* **87**: 997–1008

Barbareschi M, Weidner N, Gasparini G, Morelli L, Forti S, Eccher C, Fina P, Leonardi E, Mauri MF, Bevilacqua P and Dalla Palma P (1995) Microvessel density quantification in breast carcinomas. Assessment by light microscopy vs. a computer-aided image analysis system. *Appl Immunohistochem* **3**: 75–84

Bettelheim R, Mitchell D and Gusterson BA (1984). Immunocytochemistry in the identification of vascular invasion in breast cancer. *J Clin Pathol* **37**: 364–366

Bevilacqua P, Barbareschi M, Verderio P, Boracchi P, Caffo O, Dalla Palma P, Meli S, Weidner N and Gasparini G (1995). Prognostic value of intratumoural microvessel density, a measure of tumour angiogenesis, in node negative breast carcinoma. Results of a multiparametric study. *Breast Cancer Res Treat* **36**: 205–217

Bosari S, Lee AKC, Delellis RA, Wiley BD, Heatley GJ and Silverman ML (1992) Microvessel quantitation and prognosis in invasive breast cancer. *Hum Pathol* **23**: 755–761

Burgdorf WHC, Mukai K and Rosai J (1981) Immunohistochemical identification of factor VIII related antigen in vascular endothelial cells of cutaneous lesions of alleged vascular nature. *Am J Clin Pathol* **75**: 167–171

Chalkey HW (1943) Method for the quantitative morphological analysis of tissues. *J Nat Cancer Inst* **4**: 47

Costello P, McCann A, Carney DN and Dervan PA (1995) Prognostic significance of microvessel density in lymph node negative breast carcinoma. *Hum Pathol* **26**: 1196–1200

Curran RC and Gergory J (1977) The unmasking of antigens in paraffin sections of tissue by trypsin. *Experientia* **33**: 1400–1401

Fajardo L (1989) Special report. The complexity of endothelial cells. *Am J Clin Pathol* **92**: 241–250

Fina L, Molgaard HV and Robertson D (1990) Expression of the CD34 gene in vascular endothelial cells. *Blood* **75**: 2417–2416

Folkman J (1990) What is the evidence that tumours are angiogenesis dependent. *J Natl Cancer Inst* **82**: 4–6

Fox SB, Leek RD, Smith K, Whitehouse RM, Gatter KC and Harris AL (1994) Tumour angiogenesis in node-negative breast carcinomas – relationship with epidermal growth factor receptor, oestrogen receptor and survival. *Breast Cancer Res Treat* **29**: 109–116

Fox SB, Leek RD, Weekes MP, Whitehouse RM, Gatter KC and Harris AL (1995) Quantitation and prognostic value of breast cancer angiogenesis: comparison of microvessel density, Chalkey count, and computer image analysis. *J Pathol* **177**: 275–283

Gasparini G, Weidner N, Bevilacqua P, Maluta S, Boracchi P, Testolin A, Pozza F and Folkman J (1993) Intratumoural microvessel density and p53 protein: correlation with metastases in head and neck squamous cell carcinoma. *Int J Cancer* **55**: 739–744

Gasparini G, Weidner N, Bevilacqua P, Maluta S, Dalla Palma P, Caffo O, Barbareschi M, Boracchi P, Marubini E and Pozza F (1994) Tumour microvessel density, p53 expression, tumour size, and peritumoural lymphatic invasion are relevant prognostic markers in node negative breast carcinoma. *J Clin Oncol* **12**: 454–466

Goulding H, Nik Abdul Rashid NF, Robertson JF, Bell JA, Elston CW, Blamey RW and Ellis IO (1995) Assessment of angiogenesis in breast carcinoma: An important factor in prognosis? *Hum Pathol* **26**: 1196–1200

Hall NR, Fish DE, Hunt N, Goldin RD, Guillou PJ and Monson JRT (1992) Is the relationship between angiogenesis and metastasis in breast cancer real? *Surg Oncol* **1**: 223–229

Horak E, Leek R, Klenk N, Lejeune S, Smith K, Stuart N, Greenall M, Stepniewska K and Harris AL (1992) Angiogenesis, assessed by platelet/endothelial cell adhesion molecule antibodies, as indicator of node metastases and survival in breast cancer. *Lancet* **340**: 1120–1124

© Cancer Research Campaign 1997

Hsu SM, Raine L and Fanger H (1981) Use of avidin-biotin-peroxidase complex (ABC) in immunoperoxidase techniques. *J Histocytol* **29**: 577–580

Lee AKC, Delillis RA and Wolfe HJ (1986) Intramammary lymphatic invasion in breast carcinomas. Evaluation using ABH isoantigens as endothelial markers. *Am J Surg Pathol* **10**: 589-594

Macchiarini P, Fontanini G, Hardin MJ, Squartini F and Angeletti CA (1992) Relation of neovasculature to metastasis of non-small cell cancer. *Lancet* **340**: 145–146

Miliaras D, Kamas A and Kalekou H (1995) Angiogenesis in invasive breast carcinoma: is it associated with parameters of prognostic significance? *Histopathology* **26**: 165-169

Newman PJ, Berndt MC and Gorski J (1990) PECAM-1 (CD31) cloning and relation to adhesion molecules of the immunoglobulin gene superfamily. *Science* **247**: 1219–1222

Obermair A, Czerwenka K, Kurz C, Buxbaum P, Schemper M and Sevela P (1994) Influence of tumoural microvessel density on the recurrence free survival in human breast cancer: Preliminary results. *Onkologie* **17**: 44–49

Ogawa Y, Chung YS, Nakata B, Takatsuka S, Maeda K, Sawada T, Kato Y, Yoshikawa K, Sakurai M and Sowa M (1995) Microvessel quantitation in invasive breast cancer by staining for factor VIII-related antigen. *Br J Cancer* **71**: 1297-1301

Parums DV, Cordell JL, Micklem K, Heryet AR, Gatter KC and Mason DY (1990) JC70: a new monoclonal antibody that detects vascular endothelium associated antigen on routinely processed tissue sections. *J Clin Pathol* **43**: 752–757

Ramani P, Bradley N and Fletcher C 1990 QBEND/10, a new monoclonal antibody to endothelium: assessment of its diagnostic utility in paraffin sections. *Histopathology* **17**: 237–242

Siitonen SM, Haapasalo HK, Rantala IS, Helin HJ and Isola JJ (1995) Comparison of different immunohistological methods in the assessment of angiogenesis: lack of prognostic value in a group of 77 selected node negative breast carcinomas. *Mod Pathol* **7**: 745–752

Srivastava A, Laidler P, Davies R, Horgan K and Hughes LE (1988) The prognostic significance of tumour vascularity in intermediate-thickness (0.76–4.0 mm thick) skin melanoma. *Am J Pathol* **133**: 419–423

Toi M, Kashitani J and Tominaga K (1993) Tumour angiogenesis is an independent prognostic indicator of primary breast carcinoma. *Int J Cancer* **55**: 371–374

Van Hoef Mehm, Knox WF, Dhesi SS, Howell A and Schor AM (1993) Assessment of tumour vascularity as a prognostic factor in lymph node negative invasive breast cancer. *Eur J Cancer* **29A**: 1141–1145

Vermeulen PB, Gasparini G, Fox SB, Toi M, Martin L, McCulloch P, Pezzella F, Viale G, Weidner N, Harris AL and Dirix LY (1996) Quantification of angiogenesis in solid human tumours: an international consensus on the methodology and criteria of evaluation. *Eur J Cancer* **32A**: 2474–2484

Weidner N, Semple JP, Welch WR and Folkman J (1991) Tumour angiogenesis and metastasis-correlation in invasive breast cancer. *N Engl J Med* **324**: 1–8

Weidner N, Folkman J, Pozza F, Bevilacqua P, Allred EN, Moore DH, Meli S and Gasparini G (1992) Tumour angiogenesis: a new significant and independent prognostic indicator in early-stage breast carcinoma. *J Natl Cancer Inst* **84**: 1875–1887

*© Cancer Research Campaign 1997*